# Use of a patient decision aid for prenatal screening for Down syndrome: what do pregnant women say?

**DOI:** 10.1186/s12884-017-1273-0

**Published:** 2017-03-20

**Authors:** Maria Esther Leiva Portocarrero, Anik M. C. Giguère, Johanie Lépine, Mirjam M. Garvelink, Hubert Robitaille, Agathe Delanoë, Isabelle Lévesque, Brenda J. Wilson, François Rousseau, France Légaré

**Affiliations:** 1 Population Health and Practice-Changing Research Group, CHU de Québec Research Centre, Quebec, Canada; 2 Centre d’Excellence sur le Vieillissement de Québec, CHU de Québec Research Centre, Quebec, Canada; 3 Department of Family Medicine and Emergency Medicine, Laval University, Quebec, Canada; 4 CHU de Québec, Hôpital St-François d’Assise, and Obstetrics and Gynecology Department, Faculty of Medicine, Laval University, Quebec, Canada; 5 Department of Epidemiology and Community Medicine, Faculty of Medicine, University of Ottawa, Ottawa, Canada; 6 Department of Molecular Biology, Medical Biochemistry and Pathology, Faculty of Medicine, Laval University, Quebec, Canada; 7 Health Technology Assessment and Evidence-based Laboratory Medicine, Quebec, Canada; 8 Population Health and Optimal Health Practice Research Group, CHU de Québec Research Centre, Hôpital St-François d’Assise, 10, rue de l’Espinay/D6-730, Quebec, G1L 3L5 Canada

**Keywords:** Prenatal testing, Trisomy 21, Down syndrome, Decision aid, Shared decision making, Theoretical domains framework, Implementation, Knowledge translation

## Abstract

**Background:**

Patient decision aids (PtDAs) help people make difficult, values-sensitive decisions. Prenatal screening for assessing the risk of genetic conditions in the fetus is one such decision and patient decision aids are rarely used in this clinical context. We sought to identify factors influencing pregnant women’s use of a patient decision aid for deciding about prenatal screening for Down syndrome (DS).

**Methods:**

This qualitative study was embedded in a sequential mixed-methods research program whose main aim is to implement shared decision-making (SDM) in the context of prenatal screening for DS in the province of Quebec, Canada. We planned to recruit a purposive sample of 45 pregnant women with low-risk pregnancy consulting for prenatal care at three clinical sites. Participating women watched a video depicting a prenatal care follow-up during which a pregnant woman, her partner and a health professional used a PtDA to decide about prenatal screening for DS. The women were then interviewed about factors that would influence the use of this PtDA using questions based on the Theoretical Domains Framework (TDF). We performed content analysis of transcribed verbatim interviews.

**Results:**

Out of 216 eligible women, 100 agreed to participate (46% response rate) and 46 were interviewed. Regarding the type of health professional responsible for their prenatal care, 19 participants (41%) reported having made a decision about prenatal screening for DS with an obstetrician-gynecologist, 13 (28%) with a midwife, 12 (26%) with a family physician, and two (4%) decided on their own. We identified 54 factors that were mapped onto nine of the 12 TDF domains. The three most frequently-mentioned were: opinion of the pregnant woman’s partner (*n* = 33, 72%), presentation of the PtDA by health professional and a discussion (*n* = 27, 72%), and not having encountered a PtDA (*n* = 26, 57%).

**Conclusion:**

This study allowed us to identify factors influencing pregnant women’s use of a PtDA for prenatal screening for DS. Use of a PtDA by health professionals and patients is one step in providing the needed decision support and our study results will allow us to design an effective implementation strategy for PtDAs for prenatal screening for DS.

**Electronic supplementary material:**

The online version of this article (doi:10.1186/s12884-017-1273-0) contains supplementary material, which is available to authorized users.

## Background

Health-related decisions can be difficult when they entail multiple options that may involve risk, loss, regret, or challenges to personal life values [1, 2]. Whether or not to undergo prenatal screening to assess the risk of certain genetic conditions in the fetus, screening which has been offered for several years in most developed countries, is one of these difficult decisions [3]. Deciding about prenatal screening requires effective decision support if the decision reached is to be informed and congruent with what a pregnant woman and her partner value most [4, 5]. Consequently, it is important that health professionals provide information about screening options and their risks and potential benefits. It is also necessary to help future parents reflect about the implications of living with a child with a genetic condition, and the implications of terminating the pregnancy. This decision context is thus value-laden and highly charged [6], and a significant number of pregnant women experience a less-than-satisfying decision-making process [7]. These women are consequently at higher risk of decision regret [6].

Studies indicate that most pregnant women want to be engaged in the decision-making process about prenatal screening [8, 9]. This can be achieved with patient decision aids (PtDA), tools that help engage patients in the decision-making process [10, 11]. These tools allow people to clarify the decision point, improve their knowledge about the options that are available, increase their accuracy of risk perception, and clarify what is most important to them as well as communicate it to their health professional [11–13]. A systematic review of 115 trials of PtDAs has shown that these tools are effective for assisting the decision-making process among patients facing difficult decisions and improve decision outcomes [13]. Therefore, their potential contribution to decision-making about prenatal screening for Down syndrome (DS) is extremely valuable [14].

In recent years, a number of health organizations across diverse jurisdictions have embarked on the dissemination of PtDAs to foster patient engagement in health-related decisions [15]. However, notwithstanding the positive impacts of PtDA on patient decision-making processes and decision quality [13], their implementation in routine clinical practice is not the norm [16, 17]. Implementation strategies need to be devised to foster the use of PtDAs by pregnant women facing the routine but difficult decision about prenatal screening [18]. Consequently, we sought to identify factors influencing pregnant women’s use of a PtDA for prenatal screening for DS.

## Methods

### Study design and context

This study was embedded in a sequential mixed-methods research program whose main aim was to implement shared decision-making (SDM) in the context of prenatal screening for DS in the province of Quebec, Canada. Ethics approval was obtained from the research ethics boards of the Centre de Santé et de Services Sociaux de la Vieille-Capitale (#2013-2014-29) in Quebec, and the CHU de Quebec (#B14-02-1929).

### Participants and recruitment procedures

Prenatal care in Canada is offered by family physicians (53% of pregnancies), obstetricians/gynaecologists (45%) and midwives (2%) [19]. We wanted to maximize the diversity of perspectives by drawing from clinical sites representing different clienteles and different team approaches to prenatal care. Each kind of health professional practises differently, according to studies, and has different patient loads [20]. The different types of facility also reflect women’s differing preferences, for example birthing centers tend to attract clients who prioritize continuity of care with a midwife, while people consulting obstetricians/gynecologists prioritize availability of specialists at all times [21]. Different facilities may also reflect women’s income status [22]. We thus aimed to recruit a purposive sample of 45 pregnant women split equally between three health centers in the Quebec City region, Canada: a birthing center (services provided by midwives), a family medicine site (FMS) (services provided by family physicians) and a university hospital (services provided by obstetricians/gynecologists). A sample of at least 15 individuals per subgroup is needed to reach data saturation in this study type [12, 23].

Women were eligible if they were: a) 18 years or older; b) in their second trimester of pregnancy; c) had booked a prenatal follow-up appointment in one of the three participating health centers; d) the pregnancy was not categorized as high-risk. So that our study would not influence their decision, participating women were met by a research assistant (RA) after they had made a decision about whether or not to do prenatal screening and were invited to reflect on a hypothetical decision in case of a future pregnancy.

The project coordinator and a RA first met with health professionals from the three participating health centers during a professional meeting to explain the study process and to secure their collaboration. The administrative staff of the centers made accessible their agenda for follow-up appointments for pregnant women for every recruitment day; this enabled us to identify potentially eligible participants. Afterwards, four experienced RA approached potentially eligible pregnant women in the waiting room before their prenatal appointment. They briefly explained the project, explained that data would be anonymous and confidential, and confirmed their eligibility before inviting them to participate.

### Data collection

To conduct the interviews, we met participating women either at their homes or at the health center, depending on their preference. Before beginning the interview, participants watched a single 10-min video showing a simulated prenatal care follow-up and were reminded that the decision point was about using a PtDA or not using it; and not on making a decision to do the test or not. The participants were not given the PtDA, but saw the PtDA on the video, which showed page-by-page shots as the healthcare professional went through it. All the information provided by the healthcare professional was contained in the PtDA and participants were thus in a position to judge the nature and quantity of the information in the PtDA.

The video shows two consecutive consultations between a health professional, a pregnant women and her partner. In the first visit, the health professional gives a PtDA to the couple, explains it to them and invites them to review it at home. The health professional clarifies that the pregnant woman is facing a decision to undergo prenatal screening for DS and explains that more information can be found in the PtDA to support their decision-making process. In a second meeting, without the PtDA, the couple and health professional come to a shared decision after the health professional checks that the couple have understood the pros and cons and have thought about what is most important for them. The two consultations reflect the idea that looking at a PtDA alone is not sufficient: time is needed to digest the information. However, the video ends before a choice is made so that participants would not be influenced by a specific choice during the interview. Immediately after the video, we conducted 30-min semi-structured interviews consisting of eleven open-ended questions based on the Theoretical Domains Framework (TDF).

The TDF is comprised of 12 theoretical domains relevant to behavioral change [24–26]. The 12 theoretical domains synthesize 33 behavior change theories and their multiple specific constructs. The TDF postulates that factors influencing behavior change can be mapped onto these theoretical domains and can be used to design effective theory-based implementation interventions. Underlying these factors are salient beliefs that are amenable to change and can therefore influence behavior change. The 11 interview questions were derived from nine of the 12 theoretical domains that were most relevant to the behavior of interest (see Table 1): 1) knowledge; 2) social/professional role and identity; 3) beliefs about capabilities; 4) beliefs about consequences; 5) motivation and goals; 6) memory, attention, and decision processes; 7) environmental context and resources; 8) social influences; and 9) emotions. The three remaining theoretical domains, behavioral regulation (e.g. habits), nature of the behavior (e.g. repetitive or not) and skills (e.g. training for acquiring new skills) were felt to be less relevant to our behavior of interest and targeted participants [27, 28], who were pregnant women facing a decision that is not likely to be repeated (the fertility rate in the Province of Quebec was 1.62 in 2014) [29]. At the end of the interview, we assessed participants’ socio-demographic characteristics and obstetrical and gynecological antecedents. Interviews were audio-recorded and transcribed verbatim. Each recording had an identification code and was uploaded to our database.

**Table 1:** Questionnaire based on the Theoretical Domains constructs (translated from the original French)

Theoretical Domains	Constructs	Questions
1. Beliefs About Consequences 2. Memory, Attention and Decision Processes	Advantages	What do you think are the advantages of using a Patient Decision Aid in the context of prenatal screening for Down Syndrome?
	Disadvantages	What do you think are the disadvantages of using a Patient Decision Aid in the context of prenatal screening for Down Syndrome?
	Anticipated regret	How would you feel if you decided not to use a Patient Decision Aid in the context of prenatal screening for Down Syndrome?
3. Environmental Context and Resources	Facilitators	What would make it easier for you to use of a Patient Decision Aid in the context of prenatal screening for Down Syndrome?
	Barriers	What would make it difficult for you to use a Patient Decision Aid in the context of prenatal screening for Down Syndrome?
4. Social Influences	For/approve	Who are the people important to you who might encourage you to use a Patient Decision Aid in the context of prenatal screening for Down Syndrome?
	Against/disapprove	Who are the people important to you who might discourage you from using a Patient Decision Aid in the context of prenatal screening for Down Syndrome?
5. Social/Professional Role and Identity	Descriptive norms	What do you think people you know think about using Patient Decision Aids in the context of prenatal screening for Down Syndrome?
6. Knowledge		Up to now, what did you know about Patient Decision Aids in the context of prenatal screening for Down Syndrome?
7. Emotions		In terms of emotions, what would it feel like for you to use a Patient Decision Aid in the context of prenatal screening for Down Syndrome?
8. Beliefs About Capabilities 9. Motivation and Goals	Incentives	What incentives would you need to use a Patient Decision Aid in the context of prenatal screening for Down Syndrome?

### Data analysis

To identify participants’ salient beliefs, the first author (MELP) analyzed the full transcripts. Using a content analysis approach, she read each transcript to identify all the beliefs expressed by each participant. Then, using N-Vivo v.10 software, similar beliefs were grouped into thematic salient beliefs which were than mapped onto the nine TDF constructs. The number of participants sharing the same salient belief (henceforth referred to as “n”) and the number of quotes (henceforth referred to as “q”) referring to each salient belief were calculated. A higher “q” than “n” value meant that some participants mentioned a salient belief more than once. The most frequently elicited salient beliefs in each construct (or modal beliefs) were identified by dividing the total number of citations related to this salient belief by the total number of citations relating to all the salient beliefs mapped to that construct. Based on this calculation, for the sake of a future survey (more details in conclusion), we retained the salient beliefs whose citation frequency (“q”) represented 75% of the total number of citations, and all salient beliefs whose citation frequency was at least 20% for each construct [12]. To validate the coding scheme, a second researcher (JL) independently read all the transcripts and double-checked the tree-node. She suggested the addition of relevant categories or themes and the removal of less relevant ones, and discussed a final coding scheme with the second author. Any discrepancies were resolved through discussion with team members. The same procedure was undertaken with all identified influential factors.

## Results

### Participant characteristics

**Fig. 1:**
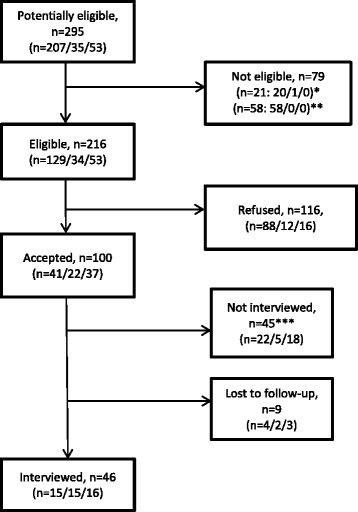
Flowchart of participants: pregnant women receiving care from obstetrician-gynecologists, family physicians or midwives

Between July 24, 2014 and January 8, 2015, out of 216 eligible pregnant women, 100 agreed to participate (46% response rate) (Fig. 1). The most frequently reported reason for declining was lack of time (see Table 2).

**Table 2:** Reasons for declining

Followed by	Lack of time	Lack of interest	Don’t like interviews	Others	Total
Ob/gyn	44	38	4	2	88
Family physician	6	5	1	0	12
Midwife	7	3	3	3	16
Total	57	46	8	5	116

Of those who accepted, we purposively selected a convenience sample of 46 participants (15 in each health center and 16 in the birthing center). Table 3 summarizes participant characteristics. Regarding the type of health professional responsible for their prenatal care, 19 participants (41%) reported having made a decision about prenatal screening for DS with an obstetrician-gynecologist, 13 (28%) with a midwife, 12 (26%) with a family physician, and two (4%) decided on their own.

**Table 3:** Sociodemographic characteristics

Characteristics*	Followed by Ob/gyn *n* = 15 n (%)	Followed by Family physician *n* = 15 n (%)	Followed by Midwife *n* = 16 n (%)	Total *N* = 46 N (%)
Age (mean ± SD)	30 ± 6	31 ± 3	29 ± 4	30 ± 4
< 35 years	12 (80)	14 (93)	15 (94)	41 (89)
> 35 years	3 (20)	1 (7)	1 (6)	5 (11)
Marital status				
Single		3 (20)	2 (13)	5 (11)
Married/common law	15 (100)	12 (80)	14 (88)	41 (89)
Employment status				
Full time	12 (80)	13 (87)	10 (63)	35 (76)
Part time		1 (7)	4 (25)	5 (11)
Unemployed and seeking work	1 (7)			1 (2)
Unemployed and not seeking work	1 (7)			1 (2)
Other	1 (7)^a^	1 (7)^b^	2 (13)^a^	4 (9)
Level of schooling				
Pre-secondary		1 (7)		1 (2)
Graduated from secondary	2 (13)	1 (7)		3 (7)
College without graduation	3 (20)			3 (7)
Graduated from college	2 (13)	1 (7)	1 (6)	4 (9)
University without graduation	1 (7)	1 (7)	1 (6)	3 (7)
Graduated from university	7 (47)	11 (73)	14 (88)	32 (70)
Household size				
2	8 (53)	8 (53)	10 (63)	26 (57)
3	4 (27)	6 (40)	5 (31)	15 (33)
4	2 (13)	1 (7)	1 (6)	4 (9)
5	1 (7)			1 (2)
Annual household income				
$15 000 to $29 999		2 (13)	1 (6)	3 (7)
$30 000 to $44 999	2 (13)	1 (7)	2 (13)	5 (11)
$45 000 to $59 999	3 (20)	3 (20)	3 (19)	9 (20)
$60 000 or more	9 (60)	9 (60)	10 (63)	28 (61)
Other	1 (7)^c^			1 (2)
Number of pregnancies				
1^st^ pregnancy	6 (40)	8 (53)	8 (50)	22 (48)
2^nd^ pregnancy	4 (27)	6 (40)	6 (38)	16 (35)
3^rd^ or more pregnancy	5 (33)	1 (7)	2 (13)	8 (17)
Health professional with whom the decision was made				
Obstetrician/gynecologist	14 (93)	2 (13)	3 (19)	19 (41)
Family physician		12 (80)		12 (26)
Midwife			13 (81)	13 (28)
Made the decision alone	1 (7)	1 (7)		2 (4)

^*^Percentages may not total 100 due to rounding

^a^Student

^b^Self-employed

^c^Don’t know (has only been in Quebec for 4 months)

### Salient beliefs

Additional file 1 presents the 54 distinct salient beliefs identified across the nine theoretical domain constructs investigated, with associated quotes. For the sake of our future survey, modal beliefs (highlighted with an asterisk* in Additional file 1) were retained according to the 75% or the 20% principle explained above (see data analysis section).

#### Beliefs about consequences

The three most frequently reported advantages of using a PtDA about prenatal screening for DS were: 1) it helps the couple reflect later together at home (i.e. helps women decide with their partners) [*n* = 25(54%); *q* = 44(27%)], 2) it is a useful source of information [*n* = 25(54%); *q* = 39(24%)], and 3) it helps make an informed decision [*n* = 25(54%); *q* = 37(23%)]. Only a few participants said the PtDA allowed them to clarify their personal values [*n* = 5(11%); *q* = 9(6%)]. The most reported disadvantage was that it could create confusion during the decision-making process [*n* = 10(22%); *q* = 15(88%)].

Further to the presentation of the PtDA in the video, some pregnant women [*n* = 14(30%); *q* = 18(50%)] said they might regret not using the PtDA if they knew it existed, but others said that they would be comfortable with the decision not to use it [*n* = 15(33%); *q* = 16(44%)].

#### Memory, attention, and decision processes

The only reported factor was that too much information was presented (i.e. information overload) [*n* = 15(33%); *q* = 17(100%)].

#### Environmental context and resources

The two most frequently reported facilitators were: 1) the PtDA should be handed out and explained by a health professional [*n* = 27(59%); *q* = 39(50%)]; and 2) the PtDA should be available on a website (for consulting online or downloading) [*n* = 16(35%); *q* = 22(28%)]. A few participants [*n* = 3(7%); *q* = 4(5%)], all of whom were followed by family physicians, reported that they might use the PtDA if they didn’t feel forced to use it because of the health professional’s limited time for follow-up. The two most frequently reported barriers were: 1) not having it to look at (i.e. lack of access to the PtDA) [*n* = 13(28%); *q* = 14(24%)], and 2) if the health professional’s presentation of the PtDA was unconvincing and failed to capture their attention or interest [*n* = 10(22%); *q* = 13(22%)].

#### Social influences

The two most frequently mentioned salient beliefs regarding who might encourage participants to use a PtDA in this context were: 1) pregnant women’s partners [*n* = 33(72%); *q* = 33(35%)] and 2) health professionals [*n* = 20(44%); *q* = 25(27%)]. The most frequently reported salient belief regarding who might *dis*courage participants from using a PtDA was also identified as the partner [*n* = 8(17%); *q* = 8(29%)], but the second most frequently reported individual was a friend [*n* = 7(15%); *q* = 7(25%)].

#### Social/Professional role and identity

The salient beliefs regarding this theoretical domain were: 1) a positive impression (i.e. a good practice that helps decision-making) [*n* = 25(54%); *q* = 29(74%)], 2) no impression at all because they didn’t know what it was [*n* = 8 (17%); *q* = 8(21%)], and 3) a negative impression [*n* = 2(4%); *q* = 2 (5%)].

#### Knowledge

Most of the participants mentioned not knowing what a decision aid was before participating in the project [*n* = 26(57%); *q* = 26(57%)], implying that knowledge of the existence of the PtDA was a significant factor influencing its subsequent use. Others said that they were given an information pamphlet by the government screening program [*n* = 17(37%); *q* = 17(37%)], and a smaller number said that they had heard of PtDAs [*n* = 3(7%); *q* = 3(7%)].

#### Emotions

The two most frequently mentioned salient beliefs regarding emotions were: 1) stress induced by knowing about risks and benefits of the test [*n* = 21(46%); *q* = 34(45%)], and 2) fear of knowing the results of the test [*n* = 9(20%); *q* = 11(15%)].

#### Beliefs about capabilities

The only salient belief mentioned regarding beliefs about capabilities was that it helps to make a decision (i.e. increases competency in decision-making) [*n* = 21(46%); *q* = 25(100%)].

#### Motivation and goals

The two most frequently reported incentives for using a PtDA for DS prenatal screening were: 1) the need to be informed [*n* = 21(46%); *q* = 24(53%)]; and 2) the presence of Down syndrome risk factors in one’s family and entourage [*n* = 6(13%); *q* = 9(20%)].

Interestingly, we had excluded three theoretical domains from the interview questions (behavioral habits, repetitiveness of the behavior and behavior skills) because we felt they were not relevant to the targeted population and behavior of interest, and in fact the women interviewed did not of their own accord mention any salient beliefs that mapped onto these three excluded theoretical domains.

## Discussion

We identified factors influencing pregnant women’s use of a PtDA for prenatal screening for DS. We recruited eligible pregnant women from three different kinds of health care center (a family medicine site, a birthing center and a university hospital) representing diverse clienteles. A total of 54 salient beliefs were mapped onto nine of the 12 domains of the TDF. Of their own accord, participants did not express any salient belief that mapped onto the three theoretical domains that we had excluded. Overall, the three most frequently-mentioned factors were: 1) influence of the woman’s partner, 2) presentation of the PtDA by a health professional and a discussion about it, and 3) lack of knowledge about PtDAs. Our results lead us to make four main observations.

First, as we observed, even if participating women identified advantages to using a PtDA about prenatal screening for DS, they also identified disadvantages, suggesting a certain ambivalence overall. On the one hand they said it provided useful information for decision-making, but they also said the amount of information could create confusion during decision-making. As reported in other studies assessing factors influencing the uptake of innovative tools in clinical practice [18, 30], we observed that a single factor may be perceived both as a barrier and a facilitator. In our case, the same factor (i.e. information giving) can be both positive (i.e. more information is helpful) and negative (i.e. too much of it is confusing). Indeed, attitude ambivalence has been shown to moderate the relationship between social norms and behaviour [31]. Research directed specifically to reducing this attitudinal ambivalence regarding information about prenatal tests could inform the improvement of PtDAs [32]. The next step would be to quantitatively assess the relative importance of these two conflicting aspects of the same influential factor to better devise a population approach to implementing PtDAs for prenatal screening for DS. Developers should clearly not overload PtDAs with information, and implementation strategies should be more individualized. In addition, in real life, the physician may not take this much time to go through the PtDA page by page, but in the second visit, as shown in the video, he/she would check that the couple understands the information and what concerns them most about the options. Decision aids alone cannot replace patient-physician interaction [33].

Second, these study results provide a more in-depth understanding of pregnant women’s opinions regarding the use of a PtDA for prenatal screening. For example, although an overwhelming majority of women mentioned that the PtDA was useful for obtaining information for decision-making, they also shared with us that knowing this information on the risks and benefits of the options would most likely increase their stress. This fear of stressful information has been reported before [34, 35]. Moreover, individuals tend to be bad forecasters: they anticipate either better or worse outcomes than can be expected based on the evidence [36]. PtDAs inform patients about potential outcomes of all options, good and bad, and help individuals better understand the probabilities associated with potential outcomes. This prompts patients to reflect then and there on risk, loss, regret, or the challenges to personal life values entailed in the decision they are facing. PtDAs raise patients’ awareness of potential outcomes they may want to avoid, which may provoke anxiety at the time of decision-making, but they also ensure that patients have realistic expectations about their choices [13, 37, 38] and give them the chance to resolve their personal uncertainties about the decision beforehand. Future implementation strategies for PtDA should therefore include managing patients’ perceptions of the anxiety they fear that using PtDA might cause, and affective support for the stress if it occurs.

Third, the most frequently reported factor influencing the use of the PtDA was the woman’s partner, with the health professional in second place. Both these findings have been reported before [38, 39]. Most of the women in this study were in a couple; in addition, the presence of the partner in the video they watched before the interviews may have disposed some of them to consider their partner as their most important influence. Perhaps the use of the PtDA also made the women aware that the decision was a values-sensitive one, in which case they would naturally want to discuss it with someone close to them (partner) or a trustworthy health professional, and have more information about it. Together these results highlight the importance of supportive relationships at the personal and professional level when making difficult health-related decisions and the need for training in this field [40]. Although promising, PtDAs alone may not be enough to ensure high quality decision-making for pregnant women and should be accompanied by training of health professionals in shared decision-making [41]. Future research on interventions should consider both the partner and the health professional in the effective implementation of PtDAs about prenatal screening for DS.

Lastly, even if one of the stated aims of PtDAs is to help patients clarify and communicate the personal values they associate with the available options [42], only a small number of participants said that the PtDA could help them identify what is most important for them and thus help them make the decision that best meets their values and their preferences. Other studies [43, 44] show that in the context of prenatal screening, one of the ethical principles is personal autonomy framed in terms of women’s choice [44], so it was surprising that only a few women mentioned the PtDA could help them clarify personal values. Further studies should emphasize the relevance of identifying and expressing personal values as a basis for good decision-making.

This study has a few limitations. First, the characteristics of our sample population do not represent all pregnant women across the sociodemographic spectrum. Women eligible for this study were highly educated and at low risk of complications or of carrying a fetus with DS (they were mostly younger than 35 years old). Although we made sure to recruit women from diverse clinical contexts, we observed few differences between sites. Second, at times it was difficult to keep to the main subject of the interview because participants focused on the behavior of undertaking the prenatal test rather than the behavior of using a PtDA to decide about the test, which was the behavior of interest of this study. This may have affected the results of our study. However, our experienced interviewers were able to redirect the conversation to the studied behavior and avoid information bias.

Third, as we mentioned, during the analysis a second researcher checked the first researcher’s tree-node. It may have been preferable for her to independently develop her own tree-node first. Nevertheless, we consider that what is most important is a systematic and thorough coding process, whether the coding is carried out by a single conscientious researcher or by a whole team of coders [45].

Lastly, although we were aiming to understand the factors influencing pregnant women’s use of a PtDA for prenatal screening for DS along the whole prenatal care pathway (i.e. during health professional visits, at home with family members or friends, and on their own), the video may have triggered only those beliefs associated with use of the PtDA during a consultation with a health professional. However, we made sure that the video included two visits with the health professional and also that participants understood they could use the PtDA outside the consultation, as was also recommended by the health professional in the video.

## Conclusion

This study allowed us to identify factors influencing pregnant women’s use of a PtDA for prenatal screening for DS. Prenatal screening decisions are difficult and pregnant women need decision support to make this values-based decision. Use of a PtDA by health professionals and patients is one step in providing the needed decision support and our study results will allow us to design an effective implementation strategy for PtDA for prenatal screening for DS. Our next step is to translate these salient beliefs into closed-ended questions to conduct a cross-sectional survey that will allow us to determine the relative importance of these beliefs and thus refine our implementation strategy. Ultimately, our goal is to ensure that all pregnant women have useful decision support tools to help them make informed values-based decisions regarding prenatal screening for DS.

## Additional file

Additional file 1: Salient beliefs. Description: The 54 distinct salient beliefs identified across the nine theoretical domain constructs investigated and their associated questions. (DOCX 23 kb)

## Abbreviations

DS: Down syndrome
FMS: Family medicine site
PtDA: Patient decision aid
RA: Research assistant
SDM: Shared decision making
TDF: Theoretical domains framework
