# Supplementary material for: Use of a patient decision aid for prenatal screening for Down syndrome: what do pregnant women say?
Source: BMC Pregnancy Childbirth. 2017 Mar 20;17:90. doi: 10.1186/s12884-017-1273-0 (PMC5359918; doi:10.1186/s12884-017-1273-0)
Supplement: Additional file 1: — Salient beliefs. Description: The 54 distinct salient beliefs identified across the nine theoretical domain constructs investigated and their associated questions. (DOCX 23 kb) [file 12884_2017_1273_MOESM1_ESM.docx]

**Additional file 1. Salient beliefs**

| **Theoretical Domains** | | **Salient beliefs** | **O/G**  **n=15**  **n (q)** | **FP**  **n=15**  **n (q)** | **MW**  **n=16**  **n (q)** | **Total**  **N=46** | | **Illustrative verbatim**  (translated from French, pregnant women code in parenthesis) |
| --- | --- | --- | --- | --- | --- | --- | --- | --- |
|  |  |  |  |  |  | **N (%46)** | **Q (%QC)** |  |
| **Beliefs about consequences** | **advantages** | -Helps couples reflect later together at home | 8 (13) | 9 (16) | 8 (15) | **25 (54)** | **44 (27)*** | “I think that it allows a better conversation between partners, because mothers are often alone in the physician’s office.” (301B) |
|  |  | -Useful source of information | 10 (14) | 8 (15) | 7 (10) | **25 (54)** | **39 (24)*** | “I can only see that it makes the information as available as possible.” (10907) |
|  |  | -Helps make an informed decision | 4 (4) | 10 (17) | 11 (16) | **25 (54)** | **37 (23)*** | “Well I think it could help make an informed decision.” (16511) |
|  |  | -Knowing advantages and/or disadvantages | 7 (10) | 8 (11) | 9 (14) | **24 (52)** | **35 (21)*** | “Yes, it was a good presentation of the advantages, disadvantages, the risks. That’s important.” (10906) |
|  |  | -Helps clarification of values | 1 (1) | 3 (7) | 1 (1) | **5 (11)** | **9 (6)** | “Also it puts your values in perspective.” (12013) |
|  |  | **N (Q)** | **30 (42)** | **38 (66)** | **36 (56)** | **-** | **(QC=164)** |  |
|  | **disadvantages** | -Could create confusion during decision-making | 2 (2) | 4 (5) | 4 (8) | **10 (22)** | **15 (88)*** | “I don’t think it helps that much for decisions. All those numbers, I think it mixes you up more than anything.” (20902) |
|  |  | -Could lead to difficult discussions for the couple | 0 (0) | 2 (2) | 0 (0) | **2 (4)** | **2 (12)** | “I think that it can cause discussions that are harder for couples or the feeling that you’re not respected in your decision.” (16502) |
|  |  | **N (Q)** | **2 (2)** | **6 (7)** | **4 (8)** | **-** | **(QC=17)** |  |
|  | **anticipated regret** | -Regret | 7 (8) | 4 (6) | 3 (4) | **14 (30)** | **18 (50)*** | “I’d say to myself, you didn’t do your homework and now look what’s happened. I would not be proud of myself.” (16502) |
|  |  | -Being comfortable with the decision not to use it | 2 (2) | 6 (6) | 7 (8) | **15 (33)** | **16 (44)*** | “Well, if I decide to not use it, it’s because I’d figured there wasn’t much chance I needed it, so I’d be good with that.” (10908) |
|  |  | -I would prefer to use it | 0 (0) | 0 (0) | 2 (2) | **2 (4)** | **2 (6)** | “But knowing that it exists, I’d prefer to have access to it.” (20202) |
|  |  | **N (Q)** | **9 (10)** | **10 (12)** | **12 (14)** | **-** | **(QC=36)** |  |
| **Memory, Attention, and Decision Processes** |  | -Too much information presented | 6 (8) | 6 (6) | 3 (3) | **15 (33)** | **17 (100)*** | “It can be too much information that’s not necessary, and that’s it.” (16511) |
| **Environmental context and resources** | **facilitators** | -Document should be handed out and explained by a health professional | 8 (9) | 8 (15) | 11 (15) | **27 (59)** | **39 (50)*** | “I think that it should be mentioned right away in the discussion, whether it’s presented positively by the doctor, or by whoever has to present any tool.” (10907) |
|  |  | -Accessibility (online or downloaded) | 6 (8) | 3 (4) | 7 (10) | **16 (35)** | **22 (28)*** | “If it’s available and known. If it’s suggested to you.” (20401) |
|  |  | -Document should be easy to understand | 4 (5) | 3 (6) | 1 (1) | **8 (17)** | **12 (15)** | “If it’s easy to understand and read. Its good with diagrams and the video, it helps you understand.” (307B) |
|  |  | -Feeling that they’re not forced to use it | 0 (0) | 3 (4) | 0 (0) | **3 (7)** | **4 (5)** | “To inform the patient without necessarily obliging her to use it. Then the patient can say what she really wants.” (11719) |
|  |  | -Time to read it during the day | 1 (1) | 0 (0) | 0 (0) | **1 (2)** | **1 (1)** | “Also having the time to do it. Because you’re often pressed for time during the day.” (301B) |
|  |  | **N (Q)** | **19 (23)** | **17 (29)** | **19 (26)** | **-** | **(QC=78)** |  |
|  | **barriers** | -Not having it to look at | 5 (5) | 2 (2) | 6 (7) | **13 (28)** | **14 (24)*** | “Yes, if it’s not available. That by itself is a barrier.” (20904) |
|  |  | -If health professional's presentation of tool is unconvincing | 1 (1) | 4 (5) | 5 (7) | **10 (22)** | **13 (22)*** | “I would say that all that matters is the way that it’s presented to you.” (12920) |
|  |  | -If the content is incomprehensible | 2 (2) | 3 (4) | 3 (4) | **8 (17)** | **10 (17)*** | “Well, if I don’t understand it. If it’s too complex.” (10908) |
|  |  | -Not enough time for the professional for presenting it | 4 (5) | 2 (2) | 0 (0) | **6 (13)** | **7 (12)*** | “For the professional, I imagine it might make the consultation longer.” (10915) |
|  |  | -Lack of information on tests available in private sector | 1 (1) | 0 (0) | 2 (5) | **3 (7)** | **6 (10)** | “The screening tests in the private sector are supposed to be 95% effective, and the public ones only 75%. Those private tests are not included.” (21006) |
|  |  | -No use if decision is already made | 0 (0) | 3 (3) | 1 (1) | **4 (9)** | **4 (7)** | “If I already have a very fixed opinion on the topic, if I know it well.” (10907) |
|  |  | -Not wanting to make decisions on one's own | 0 (0) | 3 (3) | 0 (0) | **3 (7)** | **3 (5)** | “Maybe a lack of interest and not wanting to think about it more than I have to.” (16510) |
|  |  | -If it's not approved by health professionals | 0 (0) | 0 (0) | 1 (1) | **1 (2)** | **1 (2)** | “If it’s not approved, by professionals, by the ministry or whatever, that’s a factor that would make me refuse to use it.” (21002) |
|  |  | **N (Q)** | **13 (14)** | **17 (19)** | **18 (25)** | **-** | **(QC=58)** |  |
| **Social Influences** | **for/approve** | -My partner/spouse | 11 (11) | 12 (12) | 10 (10) | **33 (72)** | **33 (35)*** | “Well, probably my spouse.” (10906) |
|  |  | -My health professional | 2 (2) | 10 (14) | 8 (9) | **20 (44)** | **25 (27)*** | “Well, the doctor who does my follow-up.” (10906) |
|  |  | -My family | 3 (3) | 6 (8) | 7 (8) | **16 (35)** | **19 (20)*** | “Well my family.” (10915) |
|  |  | -My parents | 5 (6) | 3 (3) | 3 (3) | **11 (24)** | **12 (13)** | “It would be more my mother.” (10907) |
|  |  | -My friends | 2 (2) | 1 (1) | 2 (2) | **5 (11)** | **5 (5)** | “Friends.” (16502) |
|  |  | **N (Q)** | **23 (24)** | **32 (38)** | **30 (32)** | **-** | **(QC=94)** |  |
|  | **against/**  **disapprove** | -My partner/spouse | 1 (1) | 2 (2) | 5 (5) | **8 (17)** | **8 (29)*** | “My boyfriend.” (10915) |
|  |  | -My friends | 3 (3) | 2 (2) | 2 (2) | **7 (15)** | **7 (25)*** | “Friends.” (12920) |
|  |  | -My family | 2 (2) | 2 (2) | 2 (2) | **6 (13)** | **6 (21)*** | “Maybe family members who’d say: don’t stress out about it, just trust.” (16510) |
|  |  | -Health professionals | 1 (1) | 1 (1) | 3 (3) | **5 (11)** | **5 (18)*** | “And my doctor!” (12013) |
|  |  | -My entourage | 0 (0) | 2 (2) | 0 (0) | **2 (4)** | **2 (7)** | “Sometime it’s more your entourage.” (12920) |
|  |  | **N (Q)** | **7 (7)** | **9 (9)** | **12 (12)** | **-** | **(QC=28)** |  |
| **Social/Professional Role and Identity** | **descriptive norms** | -Positive impression | 10 (12) | 12 (13) | 3 (4) | **25 (54)** | **29 (74)*** | “Yes, it’s a good practice because the goal is always that people make informed decisions. And so more information makes better decision-making.” (16716) |
|  |  | -No impression | 0 (0) | 1 (1) | 7 (7) | **8 (17)** | **8 (21)*** | “I think it’s kind of each person’s own choice.” (20901) |
|  |  | -Negative impression | 0 (0) | 1 (1) | 1 (1) | **2 (4)** | **2 (5)** | “Well, depending on your point of view, this tool could be useless.” (20804) |
|  |  | **N (Q)** | **10 (12)** | **14 (15)** | **11 (12)** | **-** | **(QC=39)** |  |
| **Knowledge** |  | -I didn't know what decision aids were | 7 (7) | 9 (9) | 10 (10) | **26 (57)** | **26 (57)*** | “But I never had tools per se.” (16509) |
|  |  | -I've seen an information pamphlet | 4 (4) | 7 (7) | 6 (6) | **17 (37)** | **17 (37)*** | “Well I received an information pamphlet.” (10908) |
|  |  | -I'd heard about them | 1 (1) | 2 (2) | 0 (0) | **3 (7)** | **3 (7)** | “Very little knowledge of it. I know that it exists, but you don’t see them much. I mean for some diseases you eventually have this kind of document.” (30004) |
|  |  | **N (Q)** | **12 (12)** | **18 (18)** | **16 (16)** | **-** | **(QC=46)** |  |
| **Emotions** |  | -Causes stress | 7 (14) | 8 (12) | 6 (8) | **21 (46)** | **34 (45)*** | “I think it caused a lot of stress.” (12013) |
|  |  | -Fear | 3 (4) | 4 (5) | 2 (2) | **9 (20)** | **11 (15)*** | “It can be a bit frightening to think you’re the person it could happen to.” (16511) |
|  |  | -Anxiety | 0 (0) | 4 (4) | 4 (5) | **8 (17)** | **9 (12)*** | “Well, for sure I could have a bit of anxiety. Anxiety! That’s another big word…” (11719) |
|  |  | -Reassuring | 0 (0) | 4 (4) | 4 (4) | **8 (17)** | **8 (11)*** | “I think it would reassure me because I would feel I had made a choice with all the necessary information.” (10907) |
|  |  | -Insecurity/fear of being responsible for bad decisions | 0 (0) | 2 (2) | 1 (1) | **3 (7)** | **3 (4)** | “There is certain insecurity because it’s just me making the decision. If I made the wrong one, it’ll be on my shoulders.” (12920) |
|  |  | -Being annoyed/angry | 0 (0) | 1 (1) | 2 (2) | **3 (7)** | **3 (4)** | “Suppose it happens to me, I think I’d be really angry with myself for not using it.” (16502) |
|  |  | -I'm not happy if the health professional does not show me the tool | 2 (2) | 0 (0) | 1 (1) | **3 (7)** | **3 (4)** | “I’d think the doctors hadn’t done their job, if they didn’t show it to us. I would not be happy.” (309) |
|  |  | -Frustration | 0 (0) | 0 (0) | 3 (3) | **3 (7)** | **3 (4)** | “If I knew that on existed, I think I’d be really frustrated not to have access to it.” (20804) |
|  |  | -I would be happy | 0 (0) | 2 (2) | 0 (0) | **2 (4)** | **2 (3)** | “Well, personally I would be happy to have it.” (11719) |
|  |  | **N (Q)** | **12 (20)** | **25 (30)** | **23 (26)** | **-** | **(QC=76)** |  |
| **Beliefs About Capabilities** |  | -Helps to make a decision/increase competency in decision-making | 7 (8) | 6 (8) | 8 (9) | **21 (46)** | **25 (100)*** | “To be able to say, well, I have an important decision to make and I have something that could help me to make this decision.” (10907) |
| **Motivation and Goals** | **incentives** | -Need to be informed | 3 (3) | 7 (9) | 11 (12) | **21 (46)** | **24 (53)*** | “Well, to know, to know the facts.” (16714) |
|  |  | -Down syndrome risk factors in my family & entourage | 3 (6) | 2 (2) | 1 (1) | **6 (13)** | **9 (20)*** | “For sure if someone has a family member with Down syndrome or other problems, the person is more likely to read it.” (11719) |
|  |  | -Chance to discuss as a  couple | 2 (2) | 1 (1) | 3 (3) | **6 (13)** | **6 (13)** | “To have a discussion with my spouse. To help us because we had a decision to make.” (20401) |
|  |  | -The health professional's approach when presenting it | 0 (0) | 2 (2) | 4 (4) | **6 (13)** | **6 (13)** | “Motivate me? Just the health professional presenting the tool, explaining what it is and what it’s for.” (20601) |
|  |  | **N (Q)** | **8 (11)** | **12 (14)** | **19 (20)** | **-** | **(QC=45)** |  |

O/G: Obstetrician/gynecologist; FP: family physician; MW: midwife; n: number of persons; q: number of quotes; N: total number of persons; Q: total number of quotes; %46: percentage out of all participants (n=46); QC: total number of quotes per construct; * : Modal beliefs.
